# Examining the Impact of a Brief Compassion Focused Intervention on Everyday Experiences of Compassion in Autistic Adults Through Psychophysiology and Experience Sampling

**DOI:** 10.1007/s10484-024-09681-y

**Published:** 2025-01-03

**Authors:** Chase S. Sherwell, Deanna Varley, Claudia Kinnane, Wesley Turner, David Zimmerman, James N. Kirby

**Affiliations:** 1 Compassionate Mind Research Group, School of Psychology, University of Queensland, Brisbane, QLD Australia; 2 UQ Learning Lab, School of Education, The University of Queensland, Brisbane, QLD Australia; 3 The Matilda Centre for Research in Mental Health and Substance Use, University of Sydney, Sydney, QLD Australia; 4 Minds & Hearts, Brisbane, QLD Australia

**Keywords:** Compassion, Compassion focused therapy, Heart rate variability, Autism

## Abstract

Autistic adults experience greater rates of anxiety and depression compared to the general population. Compassion-focused therapy interventions, aimed at promoting self-compassion capabilities, have shown efficacy in improving mental health outcomes in autistic and non-autistic samples suffering from self-criticism that contribute to difficulties in emotion regulation. We explored the experiences of autistic adults during a brief one-week online self-compassion exercise to evaluate it’s feasibility and acceptability through self-report, experience sampling, and parasympathetic activity measured via HRV. Pre- to post-intervention comparisons showed significant improvement in trait self-compassion and fears of self-compassion, but this did not extend to acute measures of psychological distress (depression, anxiety, and stress) nor difficulties in emotion regulation. HRV measures displayed significant increases during self-compassionate practice, although there were no significant changes in physiological reactivity pre- to post-intervention. Experience sampling measures found that whether participants acted on opportunities to be self-compassionate was predictive of concurrent evaluations of affective system activation, whereby acting self-compassionately was associated with greater reported activation of the soothing affective system. Together, our findings support the use of multimodal approaches to investigating the accessibility and efficacy of compassion-focused approaches to resolving emotional difficulties experienced by autistic adults.

## Introduction

There has been growing interest in mental health research into how self-compassion—the sensitivity to one’s own distress and trying to then alleviate or prevent it (Gilbert, 2014)—might aid in regulating emotional experiences and managing mental health difficulties such as anxiety and depression across a range of clinical presentations including autism (Cai et al., 2024; Galvin & Richards, 2022). Research in samples of non-autistic adults has repeatedly demonstrated that self-compassion is associated with improved psychological wellbeing (Zessin et al., 2015), better adaptive coping skills (Ewert et al., 2021), improved self-efficacy in the face of failures (Liao et al., 2021), and better psychophysiological functioning of the parasympathetic system as measured by heart rate variability (HRV; Di Bello et al., 2020). Several interventions have been developed to cultivate self-compassion (Kirby et al., 2017), with meta-analysis of studies using non-autistic samples showing such interventions lead to significant moderate improvements in self-compassion, moderate decreases in stress, depression, anxiety, and self-criticism, and large reductions in rumination (Ferrari et al., 2019). Given these established relationships, self-compassion could be an important target for clinical interventions to help autistic adults effectively manage the difficult experiences and emotions that contribute to anxiety and depression.

Autism is a lifelong neurodevelopmental condition characterised by differences in social communication, social interactions, sensory sensitivities, and restricted and repetitive interests and behaviours (APA, 2022). Autistic adults experience higher levels of anxiety and depression, as well as difficulties in emotion regulation, compared to non-autistic adults (Hollocks et al., 2019; Galvin & Richards, 2022). Self-compassion is also significantly lower in autistic adults compared to non-autistic adults (Cai et al., 2023; Galvin & Robinson, 2023; Howes et al., 2021). However, autistic adults with higher self-compassion display lower symptoms of anxiety and depression, with self-compassion mediating the positive relationship between autistic traits and anxiety and depressive symptoms (Cai, et al., 2023; Galvin & Robinson, 2023). Given these associations, researchers are increasingly interested in self-compassion as an important potential target for clinical interventions to help autistic adults effectively manage the difficult experiences and emotions that contribute to anxiety and depression.

### Compassion-Focused Interventions in Autism

Understanding how autistic adults experience and perceive compassion is critical to developing effective means of fostering the capacity for self-compassion. To this end, Cai et al. (2023) interviewed autistic adults about their experience of self-compassion, identifying three key themes. First, autistic adults perceived value in being self-compassionate, indicating that it helps build courage and made them feel better about themselves. Second, they found it hard to be self-compassionate, with the authors noting self-criticism tended to be participants’ instinctual response when making mistakes or not living up to their own standards. Finally, participants recognized the ability to be self-compassionate can build over time. Fortunately, compassion-focused interventions were initially developed to target these critical aspects: the difficulties and fears one can have with being self-compassionate, the self-criticism that is typically experienced as the core self-relating style, and fostering self-compassion as a malleable competency (Gilbert, 2014).

Cai et al. (2024) designed and evaluated a novel online self-compassion program for autistic adults in a sample of 39 autistic adults aged 20–77 years. The Aspect Self-Compassion Program for Autistic Adults (ASPAA) program included six weekly online modules co-designed with autistic researchers and community members, and informed by the evidence-based compassion-focused interventions of Mindful Self-Compassion (Neff & Germer, 2013) and Compassion Focused Therapy (CFT; Gilbert, 2014). Participants reported significant improvements in self-compassion, positive affect, and psychological wellbeing, as well as significant reductions in anxiety and depressive symptoms, and reductions in emotion regulation difficulties. However, some autistic adults reported emotional difficulties or distress when completing self-compassionate exercises.

Negative or aversive responses to self-compassion are not necessarily uncommon. The term *fears of compassion* was coined to describe dispositions, beliefs, or attitudes that lead to avoidant or fearful reactions to compassion (Gilbert, 2010). Meta-analytic research indicates that fears of self-compassion are strongly related to anxiety and depressive symptoms, as well as self-criticism (Kirby et al., 2019). CFT, developed by Gilbert (2014) directly aims to target not only self-criticism, but also the fears of self-compassion that can be experienced by people. Brief CFT interventions, delivered as self-directed and online interventions have found to significantly decrease fears of self-compassion in non-autistic samples (Kim et al., 2020), but fears of self-compassion in online interventions have not yet been examined with autistic adults as a potential resistance to effective intervention outcomes, nor as a potential mechanism of action.

### The Tripartite Model of Emotional Regulation

**Fig. 1:**
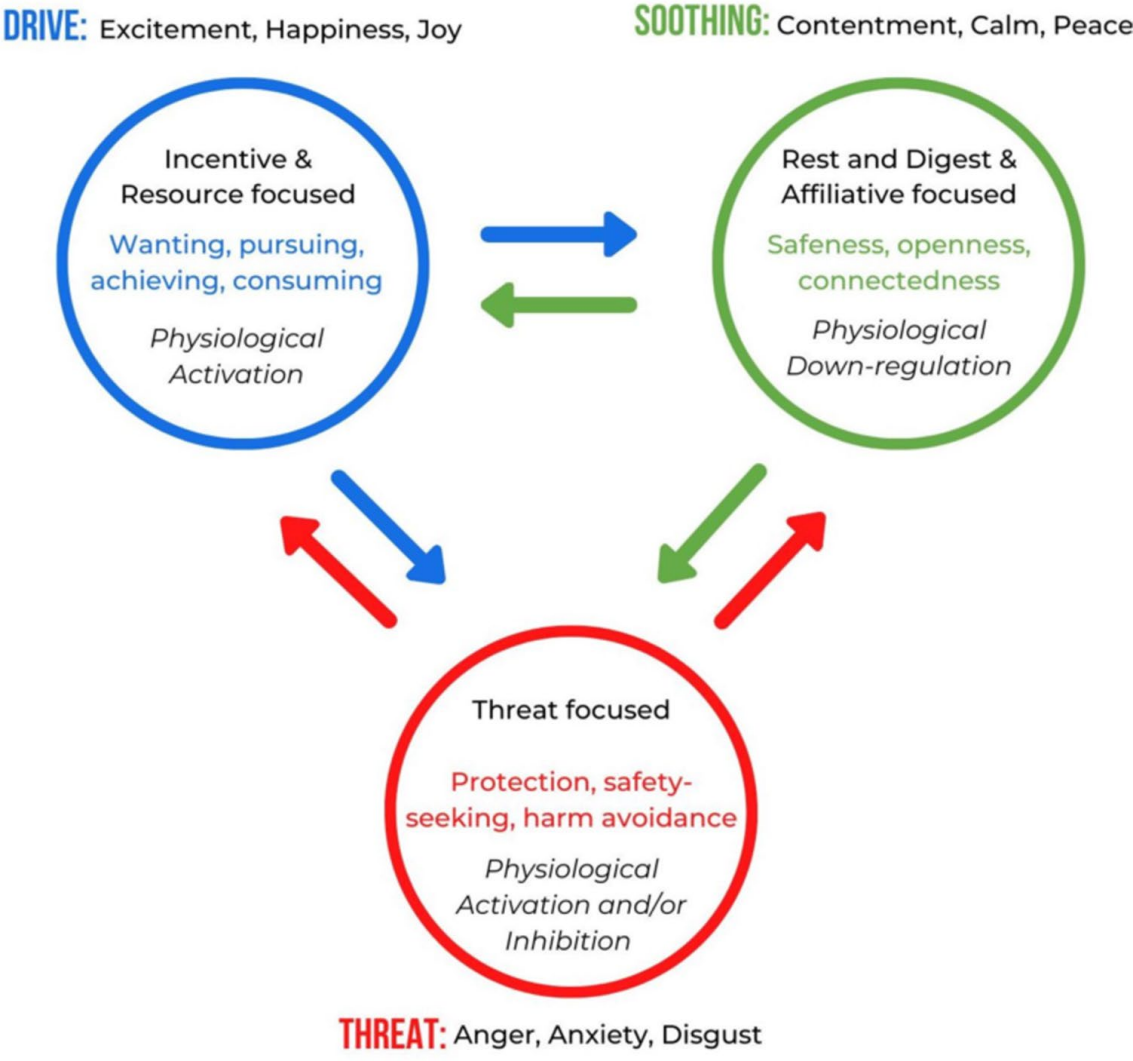
The tripartite model of emotion regulation: the three circle model. From Petrocchi, Kirby, & Baldi, Essentials of Compassion Focused Therapy: A Practice Manual for Clinicians, reprinted with permission from Routledge and Adapted from Gilbert (2005)

CFT aims to improve mental health by focusing on increasing self-compassion which helps improve one’s emotion regulation ability (Kirby & Petrocchi, 2023). According to the tripartite model of emotion regulation, there are three key emotion regulation systems, depicted in Fig. 1, which comprises the threat system (red circle), the drive system (blue circle), and soothing system (green circle). The function of the threat system is to help protect the person from danger. These dangers can be social or physical, and emotions activated in this system include anger, anxiety and fear. The function of the drive system is to support the person to pursue goals and resources, such as pursuing relationships and increased social standing, and emotions key to this system include excitement, happiness, and joy. Both the threat and drive systems are associated higher activation of the sympathetic nervous system (Petrocchi et al., 2024). In contrast, the soothing system’s primary function is to help the person rest and recover, as associated with activation of the parasympathetic nervous system (Petrocchi et al., 2024). Emotions corresponding to this system include low arousal positive emotions such as contentment, calmness and peacefulness.

Importantly, this model examines these emotional systems in relation to each other, with the soothing system helping physiologically down-regulate the activating threat and drive systems. Research in non-autistic samples indicates that over-dominant threat and drive systems predict greater levels of negative affect, greater difficulties with emotion regulation and greater symptoms of depression, anxiety and stress (Moloney-Gibb et al., 2024). The aim of CFT interventions is to help increase self-compassion, which may increase the soothing system’s emotion regulatory ability, reducing activation of the threat system. There are multiple intervention evaluations showing CFT can increase self-reported soothing emotions, as well as reduce threat emotions, and importantly increase parasympathetic physiological activity as measured by HRV (Kim et al., 2020; Matos et al., 2017; Steffen et al., 2021). Although compassion-based interventions have been shown to be helpful for autistic adults (Cai et al., 2024), the extent to which self-compassion can help stimulate the parasympathetic system in autistic adults remains unknown.

### Heart Rate Variability

HRV measures reflecting parasympathetic activity have provided valuable insights into emotional reactivity and regulation. Comparing activity at rest to that during active exposure to a stimulus or task can reflect engagement of regulatory processes, or *reactivity* (Laborde et al., 2018). Similarly, comparison of the active exposure to a subsequent resting period can reflect *recovery*. Increasingly, 3R (Rest, Reactivity, Recovery) paradigms have been used to gauge physiological responses to emotionally distressing stimuli as a measure of emotional reactivity, and in particular to measure changes in reactivity following intervention (Sherwell & Kirby, 2024). Critically, both increases and decreases in parasympathetic activity can be seen as adaptive or beneficial depending on the context (Laborde et al., 2018). Somewhat counterintuitively, greater decreases in parasympathetic activity (lower HRV) in response to experimentally induced stress or difficult emotions predicts positive mental health outcomes (Schiweck et al., 2018; Stange et al., 2017). Various authors have argued that this reflects the willingness to engage with distress, whereas reduced reactivity may reflect avoidant behaviour (e.g., Muhtadie et al., 2018; Steffen et al., 2021).

Indeed, evidence supporting this view has been demonstrated in studies investigating physiological shifts following compassion-focused interventions. Using a 3R paradigm to examine reactivity to a self-critical writing task before and after a 12-week CFT intervention, Steffen et al. (2021) found significant decreases in HRV during self-critical writing (reactivity), followed by significant increases during recovery. Critically, participants who displayed reliable increases in self-compassion after intervention showed increased HRV reactivity (Steffen et al., 2021). Even brief CFT exercises such as compassionate-self practice in the form of compassion-focused meditations have shown effects on HRV. Kim et al. (2020) used a 10-min guided imagery exercise including: (1) establishing a grounding body posture, (2) soothing-rhythm breathing, and (3) imagery focused on cultivating a sense of having a compassionate-self with qualities of wisdom, strength and commitment to be compassionate. After a 2-week period of daily practice, this brief audio-guided compassionate self-practice increased resting HRV for non-autistic adults who practiced it regularly, as well as significant increases in HRV during imagery exercise (Kim et al., 2020). Following from these results, Kim et al. (2024) replicated the finding that engagement in compassionate imagery exercise increases HRV in a sample of clinically depressed clients.

Importantly, physiological shifts in response to both stressful and soothing stimuli may be indicative of dispositional factors critical to self-compassion. Early evidence evaluating HRV responses to compassion-focused imagery, intended to stimulate the soothing affect system, showed some evidence that participants high in self-criticism and insecure attachment displayed decreases in HRV (Rockliff et al., 2008). Similarly, Baldwin et al. (2020) exposed participants to a compassion focused imagery exercises before and after being exposed to an attachment-prime task intended to enhance attachment security. Participants reporting high levels of anxious and avoidant attachment tended to show large decreases in HRV in response to the initial compassion-focused imagery task. This effect was reversed following the attachment-prime task, with participants showing increases in HRV in response to the second imagery task. As such, the direction and magnitude of HRV reactivity in response to both threatening/stressful and soothing stimuli can be indicative of emotional engagement and potential aversive reactions to compassion. Considering the qualitative reports of distress or difficulty in response to compassion or compassion focused interventions (Cai et al., 2023, 2024), HRV may provide an objective means of gauging responsivity to compassion exercises in situ.

### Capturing Everyday Experience

Self-compassion involves two key elements: first, noticing distress in oneself, and secondly, doing something to alleviate it. Yet, most research to date has relied on trait-based measurement of self-compassion which examines one’s tendency or disposition to be generally self-compassionate, for example, “*I try to be self-compassionate*” (Steindl et al., 2021). What could be more useful is to measure self-compassion as it is experienced in the moment it occurred (Varley et al., 2024).

Experience sampling is a sampling method where participants are asked to repeatedly report on everyday behaviour and experiences close to the time of occurrence while going about their daily lives (Csikszentmihalyi & Larson, 1987). Experience sampling methods enable participants the opportunity to respond more easily to whether they noticed opportunities to be self-compassionate, and whether they acted on those opportunities. Importantly, experience sampling is less susceptible to recall bias associated with traditional retrospective self-reports (Ellison et al., 2020).

Varley et al. (2024) examined self-compassionate opportunities and action over a week period with 125 non-autistic adults, who received five experience sampling surveys per day. They found that, overall, participants acted self-compassionately 72% of the time when they reported having noticed an opportunity to do so. Importantly, when individuals acted self-compassionately in everyday life, this corresponded with more positive emotional experiences, whereas if they were aware of a self-compassionate opportunity but did not act, this was associated with greater negative emotional experience (Varley et al., 2024).

Examining the experience of self-compassion in everyday life for autistic adults can provide insights into how frequently self-compassionate opportunities are noticed and acted upon. Critically, Varley et al. (2024) did not examine how self-compassionate actions impacted the individual’s tripartite model of emotion regulation. According to Gilbert’s theory (2014), acting self-compassionately should increase soothing and reduce threat-based activity in the moment it occurs, yet researchers have not yet measured this, as experience sampling methods have not been commonly used to measure self-compassion (Varley et al., 2024). Examining self-compassion and emotion regulation in everyday life with autistic adults will provide greater insights into how self-compassion is experienced and allow testing of Gilbert’s tripartite model of emotion regulation.

### Study Aims

The aim of this study was to examine how autistic adults experience self-compassion and a daily compassion-focused self-guided exercise over a one-week period for emotional wellbeing. We collected pre- and post-intervention measures to capture dispositional factors related to self-compassion including fears of self-compassion, trait self-compassion, and difficulties in emotion regulation, as well as measures of recent mental health symptomology across depression, anxiety, and stress. Before and after the intervention, participant HRV was recorded during a resting period, during a self-critical writing task, during a recovery period, and during compassionate imagery practice. The intervention involved participants being asked to engage in a daily 10-min guided compassionate imagery exercise for one week, during which they completed up to seven brief experience sampling surveys each day capturing recent experiences of self-compassion. Given the lack of empirical research examining the impact of CFT exercises for autistic adults, our study was largely exploratory and aimed to establish the feasibility of such interventions and a multimodal evaluation approach prior to larger-scale randomized controlled trials. If a self-guided exercise over one week is effective in cultivating self-compassion in the short-term for autistic adults, we would expect to see similar reductions in fears of self-compassion as shown with similar interventions in general community samples (Kim et al., 2020). We also evaluated whether participants would experience higher levels of self-compassion as well as decreases in difficulties in emotion regulation, anxiety, depression, and stress symptoms after completing the intervention. We also sought to explore how self-compassion was experienced in everyday life—specifically how acts of self-compassion corresponded to activation of the tripartite model of emotion regulation. Finally, we examined HRV activity at rest, during threat, recovery, and during the imagery exercise before and after the week of the intervention to assess potential shifts in reactivity to stressful stimuli (self-critical writing) and self-compassion practice (imagery exercise).

## Methods

This study was part of a larger pre-registered project (https://osf.io/w3ujm/). Only the measures and procedures relevant to the current research questions and aims are reported here.

### Participants

A total of 121 participants were assessed for eligibility. Interested participants completed the Autism Spectrum Quotient (AQ) and the DSM-V Self-Rated Level 1 Cross-Cutting Symptom Measure-Adult (DSM-V Q). Individuals who met the following criteria were invited to participate in this study: (1) aged 17 years or older, (2) had either a formal diagnosis of Autism Spectrum Disorder (ASD) Level 1 “Requiring Support” or Level 2 “Requiring Substantial Support” without accompanying Intellectual Impairment (according to the DSM-V-TR), or highly suspected by treating clinician and scored equal to or greater than 29 on the AQ, (3) scores on the DSM-V Q did not raise significant concerns, (4) English was their first language, (5) had not participated in compassion-focused therapy, (6) did not regularly practice mediation or mindfulness. From this pool, 94 participants did not meet eligibility criteria or declined to participate. Data from a further two participants were excluded from analysis as they did not complete all experimental tasks. The final sample consisted of 25 participants (12 Male, 13 Female) with an average age of 27.40 (*SD* = 12.20, range = 17–55). The majority (96%) identified as White and had completed high school education or higher (88%). Reported estimates of household income varied greatly across participants. See Table 1 for full demographic information. Participants received $40AUD in compensation for their time.

**Table 1:** Demographic characteristics of the sample

Characteristic	Frequency or mean	% or (*SD*)
Gender		
Male	12	48
Female	13	52
Age	27.40	(12.20)
Ethnicity	24	96
White	24	96
Other (not listed)	1	4
Education		
No diploma (5)	3	12
High school diploma (1)	10	40
Associate’s degree or diploma	3	12
Bachelor’s degree (2)	7	28
Master’s degree (3)	1	4
PhD or other doctorate	1	4
Estimated household income		
< $20,000	6	24
$20,000–$40,000	3	12
$40,000–$60,000	2	8
$60,000–$80,000	4	16
$80,000–$100,000	2	8
> $100,000	8	32

*SD* standard deviation, *$* amounts in AUD

### Measures

#### Online Questionnaires

*Demographics* Participants were asked to indicate their sex (Male, Female, or Other), age, ethnicity (White, Black or African American, Asian, Aboriginal/Torres Strait Islander, or Other), highest level of education (no diploma, high school diploma, Associate’s degree or diploma, Bachelors degree, Masters degree, or PhD or other doctorate), and an estimate of their average household income (from < $20,000 to > $100,000 in $20,000 bands).

*Fears of Compassion* To capture potential fears, blocks, or resistances to self-compassion, we used the self-compassion subscale of the Fears of Compassion Scale (FOC-S; Gilbert et al., 2011). Participants were asked to rate their agreement with 15 statements (e.g., ‘*I feel that I don’t deserve to be kind and forgiving to* myself’) on a 5-point scale from 1 (Don’t agree at all) to 5 (Completely agree). Subscale scores are calculated as the sum of all items (range = 5–75), with higher scores indicating greater fear of expressing kindness and compassion towards oneself. Internal consistency in our sample was excellent (Cronbach’s α: Time 1 = 0.907; Time 2 = 0.926).

*Self-Compassion* We used the Self-Compassion Scale (Neff, 2003) to measure self-reported capacity for self-compassion. Participants were asked to rate how often they behave in a stated manner from 1 (almost never) to 5 (almost always) in response to 26 statements (e.g., *‘I’m disapproving and judgemental about my own flaws and inadequacies’*). A total scale score is calculated by first reverse scoring the negative subscale (self-judgment, isolation, and over-identification) items and then computing the total mean across those items and those of the positive subscales (self-kindness, common humanity, and mindfulness). Total scores range from 1 to 5, with higher scores indicating greater capacity for self-compassion. In our sample, this scale showed good to excellent internal consistency (Cronbach’s α: Time 1 = 0.864; Time 2 = 0.936).

*Difficulties in Emotion Regulation* We used the brief version of the Difficulties in Emotion Regulation Scale (DERS-18; Victor & Klonsky, 2016), whereby participants were asked to rate how often a series of 18 statements (e.g., ‘*I pay attention to how I feel’*) applied to them on a scale from 1 (almost never) to 5 (almost always). Total scale scores were calculated as a sum across all items (including three reverse scored items), with a possible range of 18–90. Higher scores indicate greater difficulties in emotion regulation. In our sample, we achieved good internal consistency (Cronbach’s α: Time 1 = 0.808; Time 2 = 0.858).

*DASS* To measure symptoms of psychological distress, we used the Depression Anxiety Stress Scales (DASS-21; Lovibond & Lovibond, 1995). Participants were asked to rate the extent to which each of 21 statements (e.g., ‘*I found it hard to wind* down’) applied to them over the past week on a scale of 0 (Did not apply to me at all—NEVER) to 3 (Applies to me very much or most of the time—ALMOST ALWAYS). We calculated separate subscale scores for depression, anxiety, and stress by summing scores across the seven items of each subscale (possible range = 0–21), with higher scores indicating greater symptomology. Internal consistency was adequate or good across subscales of Stress (Cronbach’s α: Time 1 = 0.743; Time 2 = 0.815), Anxiety (Cronbach’s α: Time 1 = 0.831; Time 2 = 0.798), and Depression (Cronbach’s α: Time 1 = 0.882; Time 2 = 0.892).

#### Experience Sampling

In each experience sampling survey, participants were first asked to complete the Three Circles visual heuristic scale (Moloney-Gibb et al., 2024). This involved resizing three coloured and labelled circles to reflect the subjective experienced activation of each system (blue-*drive*, green-*soothing*, red-*threat*) over the past 30 min. As the sizing of each circle only has relational value, having no anchoring or increments, an outcome measure is calculated for each circle as the ratio of the circle diameter to the sum of all circle diameters. This provides a proportional percentage of each circle size, reflecting the ‘dominance’ of each represented emotional system, from 0 to 100%. Initial validation by Moloney-Gibb et al. (2024) in non-autistic samples found the relative reported size of each circle to be significantly, positively correlated with self-report measures of affective states corresponding to each system. Threat circle dominance was also positively associated with self-reported psychological distress (as assessed via DASS-21) and greater difficulties in emotion regulation (assessed via DERS-18), with soothing circle dominance showing opposite associations. Test–retest correlations for each circle were significant, ranging between 0.36 and 0.50.

Participants were then asked about their experiences over the previous 30 min related to self-compassion. First, participants were asked “In the past 30 min, did you have an opportunity to be compassionate to yourself?” and responded yes or no. If they answered yes, they were asked about the emotional experience related to that opportunity (“In relation to the opportunity to be compassionate to yourself, was the emotional experience you felt positive or negative?”), responding on a 7-point scale (1 = extremely negative to 7 = extremely positive). They were further asked “Did you act in a compassionate way towards yourself when this opportunity arose?”, responding yes or no.

### Heart Rate Variability

Interbeat interval data was acquired via Firstbeat Bodyguard 2 devices (Firstbeat Technologies Ltd, Jyväskylä, Finland) worn during the four experimental tasks at both Time 1 and Time 2. We used the HRVAS toolbox (Ramshur, 2010) in MATLAB (R2020b, The Mathworks Inc.) to conduct preprocessing and HRV metric calculation. First, ectopic interval detection was performed by filtering intervals exceeding 20%, 3SD from the mean, or median threshold of 4. Ectopic intervals were removed without replacement, and participant data for any condition exceeding 10% ectopic intervals was excluded from further analysis. Wavelet detrending was applied (n = 3, 6 levels) prior to calculating the root mean square of successive differences (RMSSD) and high-frequency (0.15–0.4 Hz) component (HF-HRV) for each condition separately. HF-HRV values were log transformed to better approximate normality.

### Intervention Dosage

The online guided imagery exercise was accessed via a personalised URL link that tracked visitation. We extracted the number of days in which participants accessed the exercise website as an indirect measure of intervention dosage ranging from 0 to 7.

### Procedure

Participants attended a pre-intervention (Time 1) laboratory session in which they provided informed consent prior to experimental tasks. Participants were then provided a Firstbeat Bodyguard 2 heart rate device (Firstbeat Technologies Ltd, Jyväskylä, Finland) and were instructed on how to fit the device to the chest with two electrodes. Participants were then asked to complete an online questionnaire while a baseline heart rate was recorded for 10 min. Next, participants were asked to engage in a self-critical writing task for 10 min where they were instructed to recall a time in which they experienced a failure or setback and to write about that experience with a self-critical perspective. This task has been previously used to induce significant reductions in HRV metrics argued to reflect a threat response (Steffen et al., 2021). Participants then remained seated for another 10 min and asked to either complete the online questionnaire (if not completed), or to sit comfortably. Finally, participants were asked to engage with a guided CFT imagery exercise aimed at cultivating self-compassion (described below). Participants were then free to remove the Firstbeat Bodyguard 2 device.

Following these experimental tasks, participants were instructed in how to complete experience sampling surveys as well as instructions in how to respond to the Three Circle measure. Starting the next day, participants received seven surveys per day via text message for one week. Each prompt to complete a survey required participants to respond within 20 min. Seven prompts were delivered once every two hours from 9:00am. Participants were asked to complete as many of the surveys as possible.

During this one-week period, participants were sent a daily reminder via text message at 8:00 a.m. to complete the CFT imagery exercise, or ‘meditation’ at some point during their day. Each reminder contained a link to an online audio recording of a 10-min guided imagery exercise adapted from Kim et al. (2020), designed and delivered by the last author—a clinical psychologist trained in CFT. The exercise consisted of three components: (1) body posture, (2) soothing rhythmic breathing, and (3) cultivating self-compassion. Participants were encouraged to complete this exercise at a time and place of their choosing where they could be comfortable and uninterrupted.

Following the one-week intervention period, participants returned for a post-intervention (Time 2) laboratory session, where they completed a second (identical) online questionnaire and all experimental tasks while heart rate recordings were obtained.

### Data Analysis

Statistical analyses were conducted using R (Version 4.0; R Core Team, 2021) with jamovi software (Version 2.2; The Jamovi Project, 2021) and the GAMLj toolbox (Gallucci, 2019). To examine whether the one-week intervention affected self-reported trait self-compassion, fears of self-compassion, difficulties in emotion regulation, and distress symptoms (depression, anxiety, and stress) we conducted a series of linear mixed models. Each model predicted an outcome variable with fixed factors of Time (pre- or post-intervention), dosage (number of days in which the exercise was accessed), and their interaction. A random intercept was included to account for within-subject variability.

To ensure reliable summary estimates (participant averages) from experience sampling measures, we excluded experience sampling data from participants who completed < 10 surveys. To assess whether recent self-compassionate actions were associated with different patterns of reported activity in the three emotion systems of the tripartite model, we used a series of linear mixed models with self-compassionate action (binary) predicting the relative size/ dominance of each circle (as a percentage) within samples where a compassionate opportunity was reported. A random slope and intercept were included to account for within-subject variance.

To test for differences between experimental conditions and pre-post intervention in HRV metrics, we conducted linear mixed models predicting RMSSD and log HF-HRV values with fixed factors of Time (pre- or post-intervention), Condition (baseline, self-critical writing, recovery, and meditation), and their interaction term. A random intercept was included to account for within-participant variance.

## Results

### Intervention Effects on Self-Report Measures

Initial exploration of bivariate correlations between our dependent variables (shown in Table 2) showed mostly expected relationships across related constructs. Self-compassion was inversely associated with fears of compassion and difficulties in emotion regulation at both pre- and post-intervention. Difficulties in emotion regulation were consistently correlated with fears of compassion. The relationship between these three trait measures and the three DASS subscales was inconsistent, with most significant associations observed with stress scores.

**Table 2:** Bivariate correlation coefficients between dependent variables

Variable	1	2	3	4	5	6
1. Fears of self compassion (FOC-S)	0.615** ***p* = 0.001**	− 0.736*** *p* < *0.001*	− 0.807*** *p* < *0.001*	0.490* *p* = *0.013*	*0.279* *p* = *0.117*	0.509** *p* = *0.009*
2. Self-Compassion (SCS)	− 0.639*** *p* < .001	0.606** ***p* = 0.001**	− 0.757*** *p* < *0.001*	*− 0.239* *p* = *0.250*	*− 0.201* *p* = *0.336*	− 0.539* *p* = *0.005*
3. Difficulties in emotion regulation (DERS)	0.499* *p* = 0.011	− 0.665*** *p* < 0.001	0.533** ***p* = 0.006**	*0.356* *p* = *0.081*	*0.093* *p* = *0.660*	0.604** *p* = *0.001*
4. Depression (DASS)	0.303 *p* = .140	− 0.054 *p* = .797	0.150 *p* = .473	0.579** ***p* = .002**	0.507* *p* = *.010*	0.529** *p* = *.007*
5. Anxiety (DASS)	0.271 *p* = 0.190	− 0.263 *p* = 0.204	0.186 *p* = 0.374	0.654*** *p* < 0.001	0.726*** ***p* < 0.001**	0.516** *p* = *0.008*
6. Stress (DASS)	0.337 *p* = 0.099	− 0.451* *p* = 0.023	0.453* *p* = 0.023	0.355 *p* = 0.082	0.579** *p* = 0.002	0.516** ***p* = 0.008**

Lower left triangle shows correlation coefficients between pre-intervention variables; Upper right triangle (italic) shows correlations between post-intervention variables; diagonal (bold) shows pre-post intervention correlations for the same variable. N = 25. *p < 0.05, **p < 0.01, ***p < 0.001. No correction for multiple comparisons

Table 3 below presents descriptive statistics for self-report measures pre- and post-intervention. Breaking down our sample at pre-intervention using standardized categories of symptom severity for the DASS-21, depressive symptomology was ‘normal’ for 16%, ‘mild’ for 16%, ‘moderate’ for 32%, ‘severe’ for 20%, and ‘extremely severe’ for 16% of the sample. For anxiety symptomology, 20% reported ‘normal’ severity, 12% reported ‘mild’ severity, 32% reported ‘moderate’ severity, 12% reported ‘severe’ severity, and 24% reported ‘extremely severe’ severity. Finally, in terms of stress symptomology, 12% reported ‘normal’ severity, 12% reported ‘mild’ severity, 24% reported ‘moderate’ severity, 40% reported ‘severe’ severity, and 12% reported ‘extremely severe’ severity.

**Table 3:** Descriptive statistics for self-report measures (N = 25)

Variables	Pre-intervention			Post-intervention			Pre-Post difference
	Mean (SD)	Possible range	Obtained range	Mean (SD)	Possible range	Obtained range	Mean (SD)
Fears of Self Compassion (FOC-S)	30.40 (12.60)	*5.00–75.00*	*5.00–48.00*	*24.50 (12.60)*	*5.00–75.00*	*0.00–55.00*	− *5.92 (11.00)*
Self-Compassion (SCS)	2.11 (0.45)	1.00–5.00	*1.31–3.04*	*2.36 (0.63)*	*1.00–5.00*	*1.00–4.12*	*0.249 (0.50)*
Difficulties in Emotion Regulation (DERS)	60.80 (10.30)	18.00–90.00	42.00–81.00	*59.90 (10.40)*	*18.00–90.00*	*33.00–84.00*	− *0.84 (10.00)*
Depression (DASS)	9.04 (4.95)	0.00–21.00	2.00–21.00	8.64 (5.51)	*0.00–21.00*	*1.00–21.00*	− *0.40 (4.83)*
Anxiety (DASS)	7.00 (4.65)	0.00–21.00	0.00–17.00	6.60 (4.34)	0.00–21.00	*0.00–15.00*	− *0.40 (3.34)*
Stress (DASS)	12.40 (3.78)	0.00–21.00	6.00–21.00	11.60 (4.37)	0.00–21.00	3.00–18.00	− 0.80 (4.04)

Pre-post differences values calculated as pre-intervention scores subtracted from post-intervention scores. Negative values therefore indicate decreases over time

**Fig. 2:**
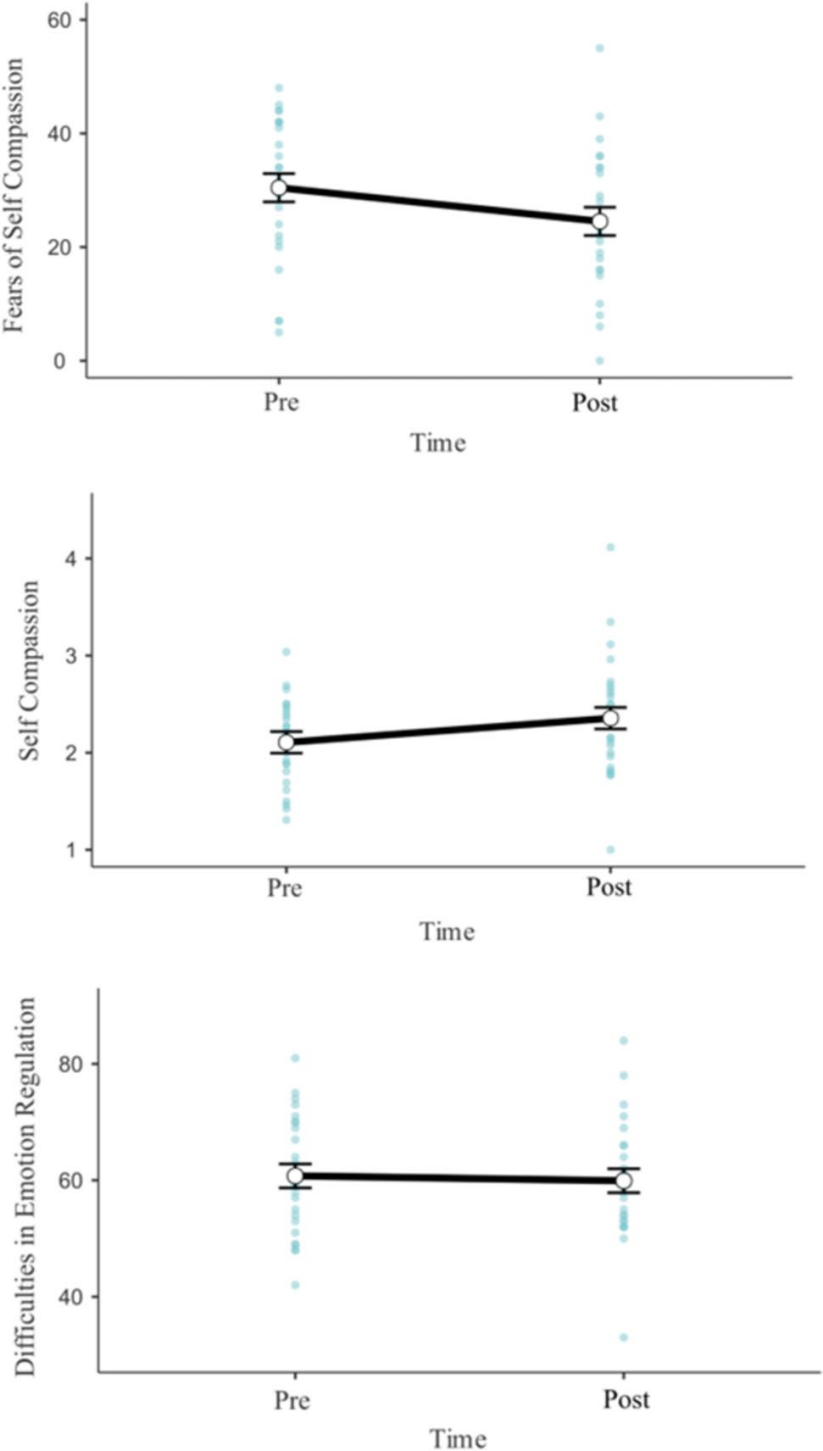
Changes in fears of compassion scale, self compassion scale, and difficulties in emotion regulation scale scores from pre- to post-intervention. Error bars indicate standard error. Blue dots represent individual datapoints

Participants completed meditation practice on an average of 3.84 (*SD* = 1.97, range = 0–7) days during the intervention period. For scores on the Fears of Self Compassion Scale, 68% (n = 17) displayed decreases from pre- to post-intervention (M = − 10.76, SD = 9.97), while 28% (n = 7) showed increases in Fears of Self Compassion (M = 5.00, SD = 3.16), with one participant showing no change over time. Linear Mixed Modelling indicated a significant effect of time, *β* = − 5.92 [CI 95% = − 10.01, − 1.83], *F*(1, 23) = 8.03, *p* = 0.009. This reflected an overall average decrease in fears of self-compassion scores of 5.92 (*SE* = 2.09) from pre- to post-intervention (see Fig. 2). There was no significant main effect of dosage, *β* = 1.17 [CI 95% = − 1.09, 3.44], *F*(1, 23) = 1.03, *p* = 0.320, nor was there a significant dosage by time interaction, *β* = − 2.07 [CI 95% = − 4.17, 0.02], *F*(1, 23) = 3.76, *p* = 0.065.

For Self-Compassion Scale scores, 64% (n = 16) of participants saw an increase (M = 0.49, SD = 0.47) from pre- to post-intervention, while 32% (n = 8) saw a decrease in Self Compassion Scale Scores (M = − 0.20, SD = 0.15), and one participant showing no change. Linear mixed modelling found a significant main effect of time, *β* = 0.25 [CI 95% = 0.05, 0.45], *F*(1, 23) = 5.88, *p* = 0.024, reflecting an overall average increase in self-compassion scores of 0.25 (SE = 0.10) from pre- to post-intervention (see Fig. 2). There was no significant main effect of dosage, *β* = − 0.006 [CI 95% = − 0.10, 0.09], *F*(1, 23) = 0.01, *p* = 0.906, nor was there a significant dosage by time interaction, *β* = − 0.004 [CI 95% = − 11.01, 0.10], *F*(1, 23) = 0.006, *p* = 0.939.

Difficulties in Emotion Regulation Scale scores did not appear to significantly differ across time or with dosage, with no main effect of time, *β* = − 0.84 [CI 95% = − 4.69, 3.013], *F*(1, 23) = 0.18, *p* = 0.673, no main effect of dosage, *β* = 1.00 [CI 95% = − 0.81, 2.81], *F*(1, 23) = 1.163, *p* = 0.291, and no significant interaction, *β* = − 1.38 [CI 95% = − 3.35, 0.60], *F*(1, 23) = 1.873, *p* = 0.184.

**Fig. 3:**
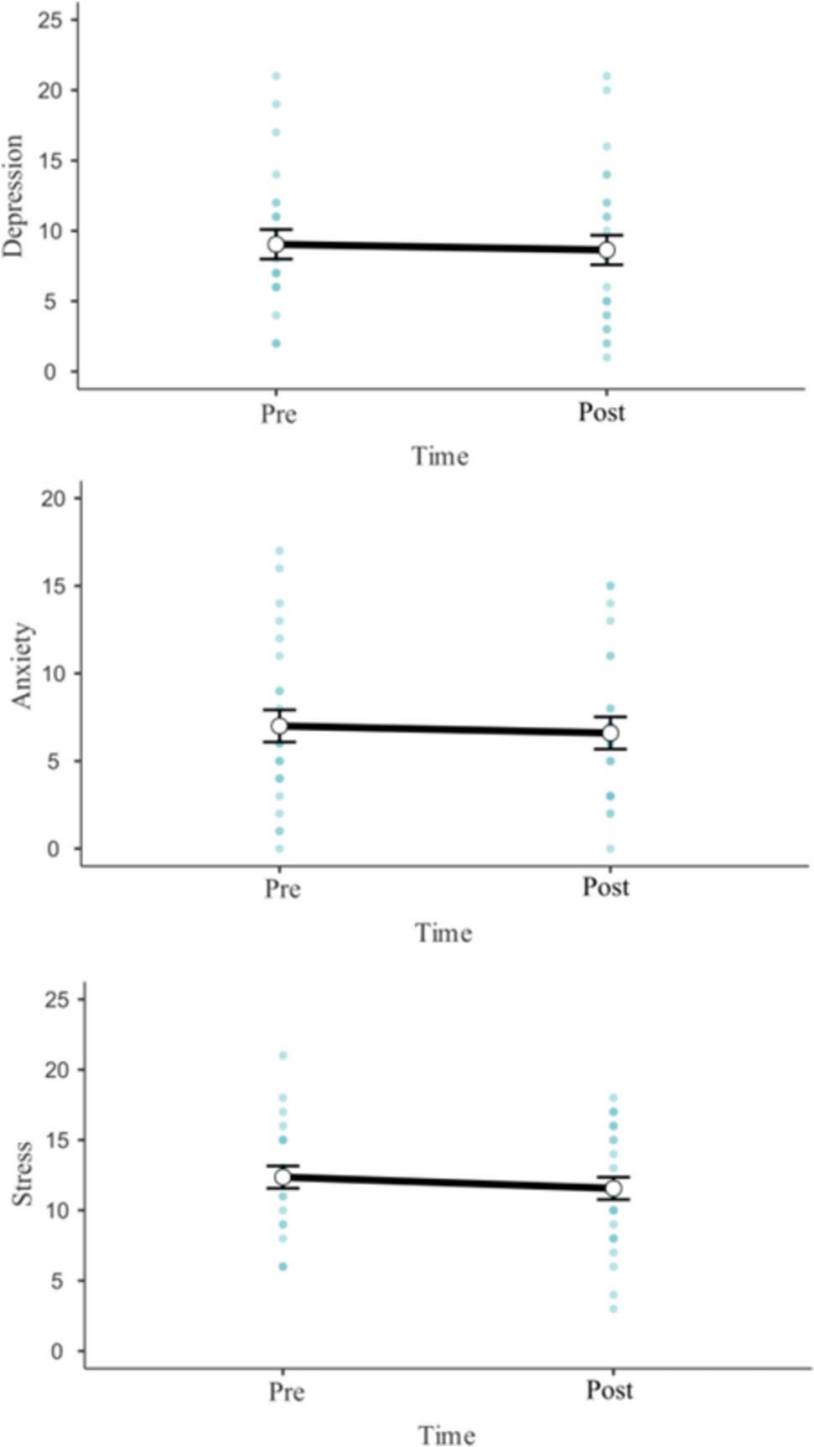
Changes in DASS subscale scores of depression, anxiety, and stress from pre- to post-intervention. Error bars indicate standard error. Blue dots represent individual datapoints

Scores on the DASS subscales of Depression, Anxiety, and Stress did not significantly differ over time or as a function of dosage (See Fig. 3). For depression scores, neither time, *β* = − 0.40 [CI 95% = − 2.20, 1.40], *F*(1, 23) = 0.19, *p* = 0.668, dosage, *β* = − 0.29 [CI 95% = − 1.24, 0.66], *F*(1, 23) = 0.36, *p* = 0.554, nor their interaction were significant, *β* = − 0.88 [CI 95% = − 1.80, 0.05], *F*(1, 23) = 3.46, *p* = 0.076. For anxiety scores, time, *β* = − 0.40 [CI 95% = − 1.74, 0.94], *F*(1, 23) = 0.35, *p* = 0.563, dosage, *β* = − 0.11 [CI 95% = − 0.99, 0.74], *F*(1,23) = 0.07, *p* = 0.798, and their interaction, *β* = − 0.12 [CI 95% = − 0.81, 0.56], *F*(1, 23) = 0.12, *p* = 0.730, were also non-significant. Stress scores also showed no significant effect of time, *β* = − 0.80 [CI 95% = − 2.30, 0.70], *F*(1, 23) = 1.10, *p* = 0.306, dosage, *β* = 0.49 [CI 95% = − 0.21, 1.19], *F*(1, 23) = 1.89, *p* = 0.182, nor their interaction,* β* = − 0.77 [CI 95% = − 1.54, 0.0007], *F*(1, 23) = 3.85, *p* = 0.062.

### Experiences of Self Compassion During the Intervention

Of a potential 49 experience sampling surveys, the participants in our sample completed an average of 29.45 surveys (*SD* = 8.15, range = 13–43). Over the 7 days of experience sampling, participants reported an average of 10.59 (*SD* = 8.56, range = 2–28) opportunities to be self-compassionate, corresponding to an average of 35.80% (*SD* = 25.91, range = 5.26–85.71) of all completed experience sampling surveys. Where participants reported an opportunity to be self-compassionate, they reported acting self-compassionately on an average of 84.98% of opportunities (*SD* = 22.55, range = 25–100).

**Fig. 4:**
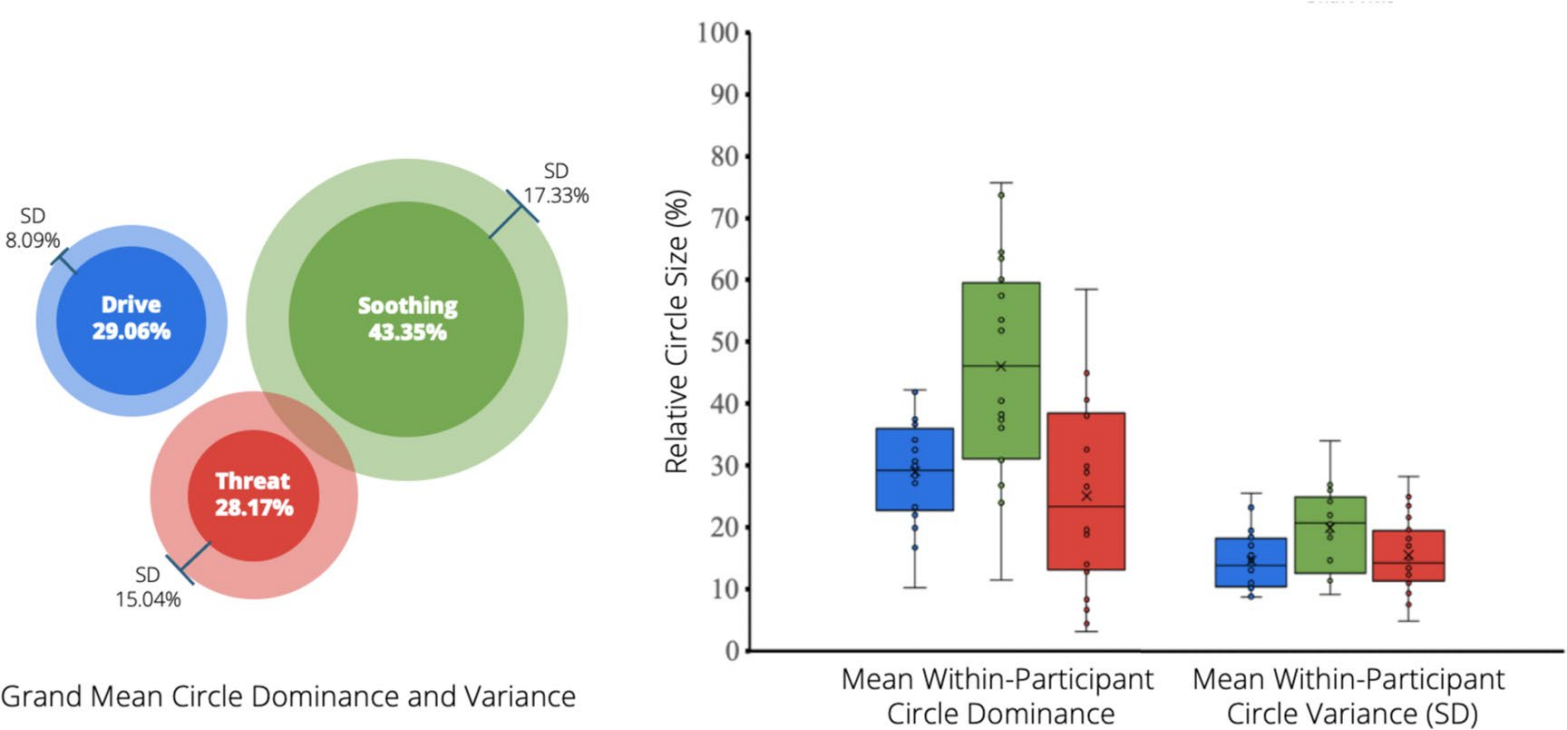
Participant (within-participant) and grand (pooled) means of relative circle size (dominance) reported across experience sampling surveys. Left panel depicts grand means of relative circle size (dominance), calculated as the mean of within-participant means, and the standard deviation (SD) across participant means. Right panel depicts distributions of within-participant circle size means, and distributions of the standard deviations (SD) within-participants to show sample-to-sample variance

Within-participant means of three circle dominance indicated soothing system activation was the most predominant system across the majority of participants (see Fig. 4). However, it was also the most variable across samples within participants, suggesting larger fluctuations over time. Soothing and threat activation displayed similar variability both within and between participants, whereas drive activation was relatively more consistent.

When participants reported an opportunity to be self-compassionate, their reported activation of motivational systems was associated with whether or not they subsequently acted on the opportunity (see Table 4). The relative size of the drive (blue) circle did not significantly differ between opportunities that were acted upon or not. Soothing (green) circle size was significantly larger when participants reported acting self-compassionately (*M* = 48.30%, SE = 3.88) compared to when they did not act (*M* = 33.60%, *SE* = 4.66). Conversely, threat (red) circle size was significantly smaller when participants acted compassionately (*M* = 21.00%, *SE* = 2.85) compared to when they did not (*M* = 40.70%, *SE* = 5.10).

**Table 4:** Summary of the effects of compassionate action on measures of emotional states

Outcome variable	Fixed effects factor	*β*	*SE*	95% CI	*t*	*p*	ICC (participant)
Drive dominance (%)	Intercept	27.72	2.28	23.26, 32.20	12.17	< 0.001	0.230
	Compassionate action	5.88	3.05	− 0.10, 11.90	1.93	0.057	
Soothing dominance (%)	Intercept	40.90	3.52	34.04, 47.90	11.62	< 0.001	0.287
	Compassionate action	14.80	4.89	5.21, 24.40	3.03	0.016	
Threat dominance (%)	Intercept	30.80	3.48	24.00, 37.70	8.87	< 0.001	0.501
	Compassionate action	− 19.70	4.47	− 28.40, − 10.90	− 4.41	0.001	
Emotional experience (1–7)	Intercept	4.16	0.14	3.88, 4.43	29.89	< 0.001	0.198
	Compassionate action	2.18	0.32	1.55, 2.81	6.77	< 0.001	

When asked to report their emotional experience in response to a reported self-compassionate opportunity, participants reported significantly more positive experiences if they acted compassionately (*M* = 5.25, *SE* = 0.13) compared to when they did not (*M* = 3.07, *SE* = 0.27).

### Physiological Activity Pre- and Post-Intervention

We successfully obtained both pre- and post-intervention HRV data from a subsample of 15 participants. We excluded data from conditions where ectopic beats were detected in < 10% of RR intervals. This removal did not alter the pattern of results, and therefore we present the data excluding these samples.

**Fig. 5:**
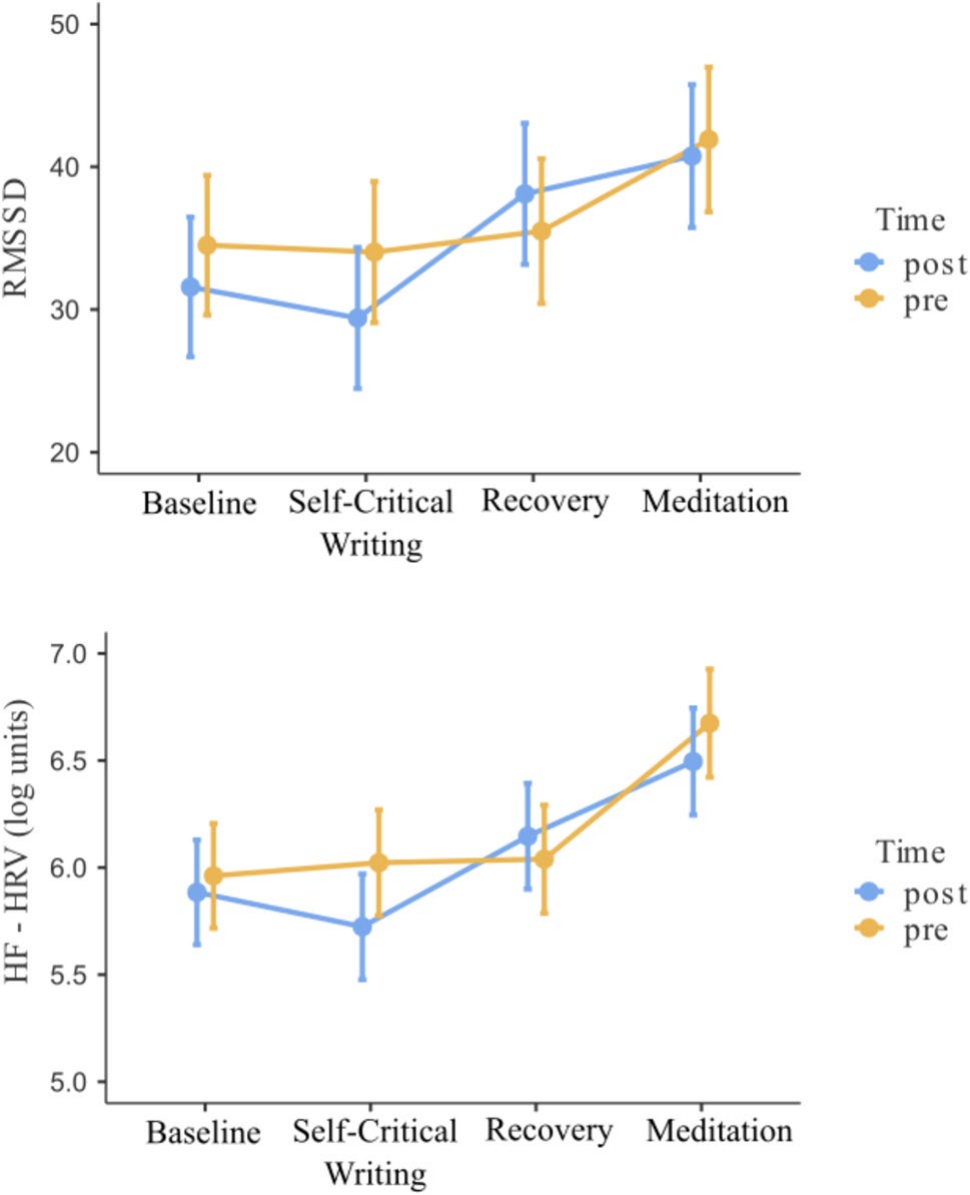
Heart rate variability metrics (RMSSD and log HF-HRV) within each experimental condition at both pre- and post-intervention. Error bars indicate standard error of the mean. RMSSD: Root mean square of successive differences. HF-HRV: High-frequency heart rate variability (log transformed)

For RMSSD values, a significant effect of condition was found, *F*(3, 87.2) = 5.28, *p* = 0.002 (see Fig. 5). Bonferroni corrected post-hoc comparisons indicated baseline RMSSD (*M* = 33.00, *SE* = 4.58) was significantly lower than meditation RMSSD (*M* = 41.30, *SE* = 4.66), *t*(87.4) = 3.17, *p* = 0.012. Further, RMSSD during the self-critical writing task (*M* = 31.70, *SE* = 4.58) was significantly lower than during meditation, *t*(87.3) = 3.64, *p* = 0.003. The main effect of time, *F*(1, 87.3) = 0.68, *p* = 0.411, and the time by condition interaction, *F*(3, 87.1) = 0.71, *p* = 0.550, were not significant.

The same model evaluating log transformed high-frequency (HF) HRV found a similar pattern, with a significant main effect of condition, *F*(3, 87.2) = 13.23, *p* < 0.001. Bonferroni corrected post-hoc comparisons indicated baseline HF-HRV (*M* = 5.92, *SE* = 0.23) was significantly lower than during meditation (*M* = 6.59, *SE* = 0.23), *t*(87.3) = 5.36, *p* < 0.001. HF-HRV was also significantly lower during the self-critical writing task (*M* = 5.87, *SE* = 0.23) compared to meditation, *t*(87.3) = 5.70, *p* < 0.001. Further, HF-HRV was significantly lower during the recovery period (*M* = 6.09, *SE* = 0.23) compared to meditation, *t*(87.1) = 3.89, *p* = 0.001. The main effect of time, *F*(1, 87.3) = 1.65, *p* = 0.202, and the time by condition interaction, *F*(3, 87.1) = 0.98, *p* = 0.407, were not significant.

**Fig. 6:**
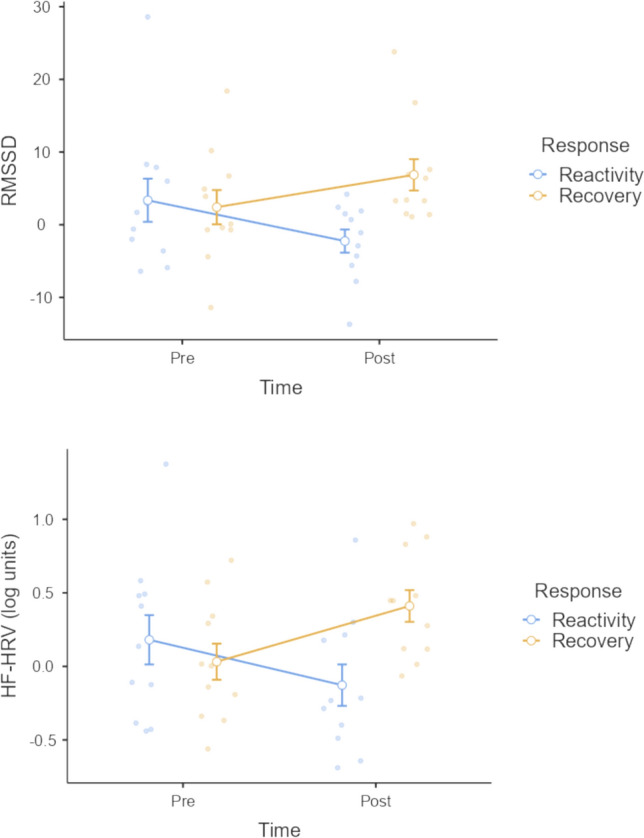
Heart rate variability (RMSSD and log HF-HRV) change scores from baseline to self-critical writing (reactivity) and from self-critical writing to recovery period (recovery) at both pre- and post-intervention. Error bars indicate standard error of the mean. RMSSD: Root mean square of successive differences. HF-HRV: High-frequency heart rate variability (log transformed). Reactivity is calculated by subtracting baseline HRV from that observed during the self-critical writing task. Recovery is calculated by subtracting HRV during the self-critical writing task and the following recovery period

As a post-hoc exploratory descriptive analysis, we inspected the reactivity (baseline HRV subtracted from self-critical writing HRV) and recovery (self-critical writing HRV subtracted from recovery period HRV) responses. Our primary goal here it to demonstrate individual differences in phasic HRV shifts across our sample. As seen in Fig. 6, HRV responses (shifts between experimental conditions) varied greatly across the sample, with participants displaying both increases (positive values) and decreases (negative values) in HRV in response to the self-critical writing task (reactivity) and following this task (recovery). At the group level, reactivity appeared to decrease from pre- to post-intervention, with many participants displaying *reduced* RMSSD and HF-HRV in response to the self-critical writing task. Interestingly, and in contrast to pre-intervention, the majority of participants at post-intervention showed *increases* in RMSSD and HF-HRV during the recovery period.

## Discussion

The aim of this study was to examine how autistic adults experience self-compassion in everyday life, how this corresponds to the tripartite model of emotion regulation, and how a daily compassion-focused intervention over a one-week period might influence HRV activity using the 3R paradigm, dispositional factors known to influence self-compassion, and distress symptomology. The results from our study were mixed. In terms of self-report measures, we saw fears of self-compassion reduced post-intervention, while self-compassion scores increased—which is the primary aim of the compassionate-self practice. However, we saw no change in symptoms of depression, anxiety or stress, which largely remained within the normal or elevated range, indicating that a brief one-week compassionate-self exercise may not be strong enough to significantly decrease symptoms of depression, anxiety or stress. Further, there was no change in scores for difficulties in emotion regulation, again suggesting that the intervention may have been too brief. Finally, dosage had no significant impact on any of the outcome measures.

When it came to experiences of self-compassion in everyday life, participants reported acting on opportunities to be self-compassionate 82% of the time, which is slightly higher than the rate of 73% observed in a non-autistic sample (Varley et al., 2024). Critically, we found that when participants did act self-compassionately, they experienced significantly greater soothing system activity, and reduced threat-based activity, supporting our hypothesis and Gilbert’s model of self-compassion and the tripartite model of emotion regulation.

In relation to physiological activity, we replicated previous findings whereby engaging in compassionate imagery exercises increased HRV metrics (HF-HRV and RMSSD) compared to baseline (Kim et al., 2020, 2024). Participants responded to the compassion-focused imagery exercise at both pre- and post- intervention with increases in parasympathetic activity, suggesting that, on average, participants did not have aversive reactions to the compassion task. HRV reactivity to the self-critical writing task was non-significant at both timepoints, indicating a lack of reliable shifts in physiology in response to this manipulation. Further, no reliable change in reactivity was found from pre- to post-intervention. The lack of reliable reductions in HRV metrics in response to the self-critical writing task might be explained by several alternatives worthy of further exploration.

As there were no significant reduction in HRV metrics during the self-critical writing task compared to baseline as expected, this manipulation may not have successfully induced a threat response. While similar tasks have previously been successful in eliciting HRV reductions (Steffen et al., 2021), these have typically been in participants presenting with clinical mood disorders which may increase the sensitivity to self-critical threat. In our sample, we saw diverse responses to the self-critical writing task including both increases and decreases in HRV. The lack of consistency in HRV reactivity may reflect a general disposition in our sample of participants that favours avoidance of difficult emotions—we believe this is unlikely, considering our sample scored within expected normative ranges on fears of self-compassion and difficulties in emotion regulation. Future research with larger samples may resolve these discrepancies by examining the potential moderating effects of trait self-compassion and fears of compassion in the physiological response to threat. Further, development of suitable stress and/or threat induction methods for specific populations such as autistic adults, targeting specific difficulties in emotion, is needed to further research in this field.

The key finding from this research is that when autistic adults acted self-compassionately, this was associated with more positive emotional experiences and greater reported soothing regulation. These insights were possible due to the use of using experience sampling methods and having a digital interactive measure of the tripartite model of emotion regulation that helped identify how self-compassion is experienced in everyday life. Moreover, the aim of this research was not to examine the clinical efficacy of CFT, rather to examine how a specific exercise, compassionate-self practice, might influence self-compassion, emotion regulation, and mental health outcomes. It is clear from our research that a daily practice over one week is insufficient to bring about shifts in mental health outcomes using self-report scales such as the DASS and the DERS. However, the observed immediate improvements in fears of compassion and self-compassion may facilitate further improvements in clinically relevant mental health outcomes in the long-term. Future research should examine the practice over a longer-time period, for example a minimum 2-week period, such as in Kim et al. (2020). The generic practice of compassionate-self imagery was not especially tailored to the unique needs of autistic adults, which may have presented potential barriers to intended outcomes. However, our results indicate that autistic adults responded positively to self-compassionate exercises both in the laboratory, as seen in increases in HRV, and in everyday life, as seen in improvements in fears of self-compassion and trait self-compassion.

## Conclusion

Our study has displayed the advantages of a multi-modal approach to better understand the various impacts brief compassion focused exercises, such as compassionate imagery, can have for autistic adults. However, our results should be interpreted with caution due to the lack of a control group—a limitation also present in other previous studies investigating the effects of self-compassionate exercises in samples of autistic adults. While this study focused on quantitative methods to examine the feasibility of a multimodal approach to investigating interventions in Autistic adults, there would be significant benefits to incorporating qualitative methods in future work. Capturing the unique experiences, language, and challenges of Autistic adults has led to valuable insights for optimising interventions for this population (Edwards et al., 2024). Critically, we have shown that autistic adults benefit from even brief self-guided self-compassionate practice in terms of short-term benefits in trait self-compassion and aversions to self-compassion. While we did not observe significant shifts in parasympathetic reactivity in response to a brief one-week intervention, we found that self-compassionate practice induced significant increases in parasympathetic activity—suggesting autistic adults did not experience aversive responses to this component of CFT. Together, these findings support the use of self-guided practices to cultivate self-compassion in autistic adults. Further research utilising objective measures of intervention reactivity via HRV can provide critical insight into the mechanisms of action underlying compassion-focused interventions.

## Data Availability

De-identified data reported here is provided on the open-science framework registration for the described project.
